# Is Syphilis Transmitted Directly from the Father to the Child?

**Published:** 1892-01

**Authors:** C. O. Weller

**Affiliations:** Austin


DANIEL'S

### TEXAS MEDICAL JOURNAL.

A Representative Organ of the Medical Profession, and an Exponent of Rational Medicine; devoted to the Organization, Advancement and Elevation of the Profession in Texas.

PUBLISHED MONTHLY.—SUBSCRIPTION $2.00 A YEAR.

Vol. VII. AUSTIN, JANUARY, 1892. No. 7.

### Original Articles

CONTRIBUTED EXCLUSIVELY TO THIS JOURNAL.

*The Articles in this Department are accepted and published with the understanding that we are not responsible for, nor do we indorse the views and opinions of the writers by so doing.*

---

For Daniel's Texas Medical Journal.

### IS SYPHILIS TRANSMITTED DIRECTLY FROM THE FATHER TO THE CHILD?

BY C. O. WELLER, M. D., AUSTIN.

Read at the meeting of the Austin District Medical Society, December 17, 1891.

THE study of syphilis is interesting, not only on account of the influence of this disease on the health of the human family, but also on account of the obscurity that attaches to the nature of its infective contagium: which, though intangible, and I may also add invisible, is none the less real, and whose power, though hidden in its action, is so potent in its effects. One division of the general study of this disease, that of syphilis in the embryo and new born children, and the manner in which the ovum of the female is infected, has engaged the attention and diligent study of the medical profession for centuries, and at the present day it is no less a subject of earnest inquiry than it was when Paracelsus, in 1529, declared syphilis an hereditary disease, and transmissible from father to child. There have been, and are now many intelligent practitioners, however, who deny the probability, and some even the possibility, of its transmission from father to child by the spermatozoa, and hold the mother alone responsible for the direct infection of the embryo. So far, however, as my researches have gone, I have failed to find any argument or statement of supposed facts, strong enough to shake my faith in the power of a syphilitic man to procreate a syphilitic child by a healthy woman. Consequently, I answer in the affirmative the question for discussion: Is syphilis transmitted directly from the father to the child? As the nature of the syphilitic poison is not yet positively determined, though the general inclination of professional opinion is in the direction of a micro-organism, I can not do better, perhaps, than quote from the article on syphilis, written by Dr. Hyde, in the 6th volume of the Reference Hand-book of the Medical Sciences. He says: "Syphilis is a specific, infectious disease capable of transmission by inheritance, and also by the medium of fluids furnished by the pathological tissues of diseased individuals. These fluids when isolated may be said to embody all the power and potency, of the diseases and hence are described as virulent, or containing the virus of syphilis. This virus or contagious element of the secretion, has been the theme of much discussion. Its very existence has been doubted by some. Chemical analysis has failed to isolate it. Salisbury, Lastofer and others have thought they discovered the germs of the disease in the secretion, but failed to make good their pretensions. Later Pesanerski, Klebs, Bermann, Aufrect, Morison, and Birsch Herschfeld have discovered, in fluids of this kind, microccoci, which by them were supposed to be etiological factors of the disease. Finally, on the 12th of November, 1884, Lustgorten announced to the Vienna Society of Medicine, that he had recognized the bacilli of syphilis. Dotrelepont, Klempeur and others have verified these observations, but it is held by Cornil that the microbe supposed by Lustgarten to be the essential element of the syphilitic virus was indistinguishable in more than one-third of all chancres examined by him and his collaborators. While Koebner asserts that though, these organisms are recognized in most of the genital lesions of syphilis, they are not found in buccal and other extra genital lesions of the same disease. Lastly, it is admitted, that the essential conditions requisite to establish the fact, that any micro-organism is the sole factor in syphilis have never been satisfactorily met. These are: The fairly constant demonstration of the baccili in lesions of syphilis, whether occurring in one or another region or viscus of the body; next, the reproduction of generations of these germs in sterilized culture fluids; lastly, the infection of sound human beings by the medium of the latter, and the production in such infected subjects of a typical syphilis capable of transmission to a second individual by the usual methods. Though unable to declare to-day, that this or that specific germ is the essential factor in the disease, the remarkable advance of knowledge on the subject of bacterial affections, gives us a basis sufficient to establish the belief that syphilis belongs to the class of infectious granulomata; and is the result of the introduction of a parasite into the human economy." Frankel, in his Text-Book of Bacteriology, speaking of the syphilis bacillus, says: "If important factors are wanting in Lepra to positively prove bacilli the cause of the disease, this is still more the case in another disease, which somewhat resembles tuberculosis and lepra, namely, syphilis." As everybody is aware, and as experience abundantly shows, syphilis is a very infectious and easily transmissible disease. But we know hardly anything about the sources of its infectious nature, and the causes of its peculiar phenomena. Although we might be inclined to suspect a bacterium as the bearer of its virus, yet our knowledge is not sufficient to enable us to prove the correctness of such a proposition." It is thought by some that the real infective agent is the ptomaines excreted by the supposed syphilis bacilli in their growth and development. Be this true or not, or be the infective agent what it may, we do know, there is a syphilitic poison, which once introduced into the blood of a human being, subjects to its baneful influence the whole organism. That the blood is charged with this poison, has been proved by the successful inoculation of healthy persons with it. We know also that a great quantity of the poison is not necessary to the propagation of the disease. It is only requisite that the smallest conceivable particle of the virus be applied to an absorbing surface to cause the infection of a healthy individual. It has the power of appropriating the food with which it is provided by the blood and tissues, and reproducing itself indefinitely. I will state in this connection that the physiological act of fecundation is effected by the entrance of the spermatozoa into the interior of the ovum through the vitelline membrane, and their union with the substance of the vitellus. After their arrival in the interior of the vitellien cavity the spermatozoa disappear as distinctly organic elements. Their substance unites with that of the vitellus, and thenceforward the fecundated egg consists of material derived from both the male and female organisms. The greater portion of this material is that produced by the female; but that which is supplied from the seminal filaments of the male is equally essential for the production of the embryo.

The offspring, accordingly, may exhibit resemblances to either or both of the individual parents, since it originates from a union of both the generative products. Again, "This seminal fluid that impregnates the ovum is capable of giving to the developing offspring all the characters in features, size, mental disposition and liability to disease, which belongs to the father." I quote the above from Dalton & Kirkes, to remind you of the capabilities and possibilities of this little microscopic spermatozoon, and to show that the part enacted by it in the act of fecundation, and subsequent development of the embryo, is just as important as that of the ovum of the female. It not only quickens into activity forces lying dormant in the ovum, and furnishes material for the physical growth of the new being, but it also leaves its impress upon the physiological action of the different organs, and transfers, in some mysterious way, the mental characteristics of the father to his offspring. Such power being vested in the spermatozoon of a healthy man, why may we not believe that when the blood of the father is saturated with the syphilitic poison, and from that blood is secreted the spermatic fluid containing the spermatozoa they may not be so inflicted as to transmit the disease to the ovum it fecundates ?

To substantiate the affirmative of the subject for discussion, I will not go into the tedious detail of individual cases, but will quote from the writings of a few eminent authors and practitioners, whose vast experience, great ability, and honesty of observation, render them worthy of credence, and give their testimony the weight of authority. In Gross' Surgery we find the following: "In the great majority of cases of infantile syphilis the disease is communicated either through the seminal fluid of the father in the act of procreation, or by the mother through her blcod, after the ovum has taken up its residence in the uterus." That the contamination may occur in both these ways has been incontestably proved by numerous observations, conducted with such care as not to admit of any reasonable doubt. The semen is a living fluid, and in a man laboring under constitutional syphilis, the probability is that every spermatazoon is completely impregnated with the specific poison; hence, it is only necessary that it should be mixed with the material provided by the mother in order to produce thorough vitiation of the new being."

Niemeyer, alluding to the almost constant premature death and abortion of the fœtus, when a woman suffering from secondary syphilis becomes pregnant, or when a healthy woman is impregnated, and subsequently contracts the disease, says: "Inasmuch then as constitutional syphilis of the mother exerts so pernicious an influence npon her offspring that it usually perishes, either long before, or else during birth, it will be readily understood that the majority of cases of congenital syphilis, which become the object of clinical investigation and medical treatment are the progeny of syphilitic fathers. It is an extraordinary but well established fact, that syphilis may be thus transmitted from father to child without inflicting the mother, who bears the infected offspring in her womb." Dr. Hyde says, "The father alone, when the victim of an active constitutional syphilis, is capable of transmitting the disease to his child without infection of the mother. It is probable he does not possess this power before the disease is actively displayed in his own person by constitutional symptoms. That a syphilitic child may be infected by inheritance from the father alone, the mother remaining sound, is demonstrated by numerous facts. Dr. Taylor, of New York, and myself have, in this country, substantiated by reported cases the facts set forth by Kassowits and others." Prof. Zeigler asserts, "If a child is procreated while the infection lasts the disease may be transmitted to it from the mother's side as well as from the father's." In Wyeth's Surgery we find the following: "The germs of syphilis are transported within the spermatic elements, and with such potency that the impregnated ovum is affected."

Prof. Wyeth further says: "Seminal fluid from a syphilitic man, in any of the stages of the disease, is held not to be directly contagious. However, the mother may acquire the disease from the child in utero, the child being syphilitic from spermatozoon." Fournier says the effects of hereditary syphilis by paternal transmission is more disastrous than by maternal, because the percentage of disease in man is vastly greater than in woman. Out of 500 syphilitic families, 487 men and thirteen women were affected. The father may not only infect the child, but may infect the mother through the child."

Congenital syphilis may be derived from the father alone, from the mother alone, or from both parents. Paternal heredity is by far most common; the number of cases of maternal heredity is comparatively small. Maternal heredity is, however, more virulent than that derived from the father, while mixed heredity represents the greatest intensity of the disease, as manifested in the child. In regard to the mortality of hereditary syphilis; when derived from the father alone 28 per cent of the offspring perish, when from the mother alone 60 per cent.; when from both the parents, 68.5 per cent. Fournier further states, "That the inoculation experiments of Cospari and Newmann proved conclusively that the apparent immunity of the mother, who has borne a child syphilitic by its father, against the contraction of the disease from her offspring is due to the fact that she has already been infected by syphilis during the intra-uterine period of the child's life. Thus conceptional syphilis is to be classed with the hereditary form of the disease since there is no primary lesion, and it is received by the mother from the fœtus infected by the father." Prof. Loomis testifies "That syphilis may be inherited from either the father or the mother." W. London Strain, in the Glasgow *Medical Journal*, considering the subject of syphilis and marriage, and the effects of syphilis on the offspring of marriage, divides his subject into five classes. Class one, consists of cases in which both man and woman had contracted syphilis by chancre. Class two, where only the woman had had chancre and the man was sound. Class three, in which only the man had chancre and the woman was infected through the fœtus. Class four, cases in which the man had had chancre, and the woman had no manifestations of infection. Class five, cases with a suspicion of hereditary supplies." In commenting on these cases, he says with reference to classes three and four (the only two of the five that have any bearing on our subject), in which the man had had chancre, the power of conception seems to be very little affected, but the result on the product of conception is still very serious. Abortions and miscarriages are very frequent, as well as children born dead, or who die soon after birth.

I could quote similar testimony from Bumstead, Didoy, and others, but have made quotations sufficient for my purpose, and will briefly notice the argument of a very plausible writer on the negative side of this question, namely, Dr. Otis. He says that "the contagion of syphilis is always effected by the contact of a degraded cell with a healthy germinal cell. In a strict sense, then, it is always localized; cells thus degraded are practically emasculated; their capacity for usefulness is lost; of necessity, then, growth of living tissue occurring, it must take place through the normal cell elements, that is, through those which have escaped this degradation. It is plain, then, that only a portion of the germinal material of a living organism can be affected by syphilis. Sufficiently germinal material to carry on the processes of life and growth must escape, or growth would be at once arrested, and life would cease. The possibility of involving in the syphilitic dyscrasia, so infinitesimal a portion of a spermatic organism as would still enable it, in conjunction with the ovum, to play an efficient part in the growth and development of the human embryo is scarcely conceivable. Especially difficult shall we find it to accept such a view when we consider that once in connection with the ovum the syphilitic influence would be rapidly imparted to the germinal elements furnished by it. We may, then, consistently throw the great improbability of continued growth (or, indeed, of any growth) under such an unfavorable influence, into the scale with the clinical claims of those who deny the possibility of the embryo or fœtus being infected by the spermatozoa. With this view of the subject, the onus of hereditary transmission is necessarily thrown upon the mother under all, even under apparently contradictory circumstances. Hence, when an embryo, or fœtus, is the subject of syphilitic infection, we may conclude that it is the result of contact of its normal formative or germinal cells with cells degraded through the syphilitic influence furnished by the nutritive fluids of the mother, either directly through the circulation or through degraded cells from the organism gaining access to that of the embryo or fœtus by their amæboid power."

In reply, I must say I cannot agree with Dr. Otis in his affirmation that every cell that comes under the influence of the syphilitic virus is practically emasculated or devitalized, and consequently rendered totally unfit for the purposes of nutrition. If this were the case, the inevitable doom of every syphilitic would be death, for when the virus had once entered the blood current, its capacity for furnishing nutritive material to the organs and tissues of the body would be totally destroyed, and the vital processes would of necessity cease. Observation, however, shows us this is not the case. That the blood, in the active stage of syphilis, is thoroughly infected, is evidenced by the successful inoculation of healthy persons with the blood of syphilitic patients. We have no proof that a portion of the syphilitic's blood is infected, and hence incapable of nutrition, whilst another par t has escaped the influence of the virus, and only contains food suitable for nourishment. We are compelled from the evidence before us to conclude that the whole of the blood—plasma and corpuscles—is infected, yet *not* devitalized. This view of the contamination of the blood is sustained by Dr. Otis' own statement, that when a cell degraded by the syphilitic virus comes in contact with a healthy cell it is at once infected, so that when one blood cell is infected by the syphicitic virus it at once imparts the infection to the one in contact with it, and thus it proceeds until the whole mass of the blood is poisoned. Yet notwithstanding this is the case, the vital processes of the syphilitic are still performed. The blood being thoroughly saturated with the virus, it is not difficult to appreciate the fact that semen secreted from this blood partakes of its infection, whether the infective agent be a micro-organism, ptomaine, or something else. He further says: "The possibility of involving in the syphilitic dyscrasia so infinitesimal a portion of a spermatic organism as would still enable it, in conjunction with the ovum, to play an inportant part in the growth and development of the human embryo, is scarcely conceivable." I see no difficulty here. Dr. Otis' error is in supposing that every infected cell is deritalized, and the small size of the spermatic cell would not admit of its infection without its emasculation. The physiological action of the different organs is performed by cells as small, and even smaller than the spermatic cell. The infection of any organ by a virus is but the contamination of the several cells of which it is composed, and as any one or all of the many organs of the body may be infected by the syphilitic virus without being devitalized, so may the spermatozoon be poisoned by this virus, whatever it may be, and yet retain its power of penetrating the ovum, arousing into activity its latent power, bringing into life a new being, and at the same time imparting to it the infection it bears. I contend, then, that the spermatozoon, although in-infected with the syphilitic poison is not dead, and consequently rendered unfit for its functions, any more than is the blood from which it is secreted. If Dr. Otis' theories were correct, there could never be a living embryo as a result of copulation, if either the man or woman were syphilitic. His rule applies as well to the woman as to the man,—to the ovum as well as to the spermatozoon. If the spermatic cell of the syphilitic man is so degraded as to be practically emasculated, and its capacity for usefulness lost, the ovum of a syphilitic woman must of necessity be likewise affected.

Is syphilis transmitted directly from the father to the child? A proper solution of this problem is of the greatest importance. It is one concerning which every practitioner should have a decided and intelligent opinion. Upon its decision depends the propriety of a syphilitic man's marriage. In order to arrive at a proper conclusion, we should not be influenced by theoretical speculation, but submit to the arbitrament of facts, not only as observed by ourselves, but as noticed, collated and recorded by those who have made this disease a special study.

